# Anesthetic Challenges During Whole Lung Lavage: A Case Report

**DOI:** 10.7759/cureus.33659

**Published:** 2023-01-11

**Authors:** Prasanta K Das, Krishna Prasanth Yadavilli, Aswathy Girija, Subho Sarkar, Afshan Shaik, Manoj K Panigrahi

**Affiliations:** 1 Anesthesiology and Critical Care, All India Institute of Medical Sciences, Bhubaneswar, Bhubaneswar, IND; 2 Anaesthesiology, All India Institute of Medical Sciences, Bhubaneswar, Bhubaneswar, IND; 3 Pulmonary Medicine & Critical Care, All India Institute of Medical Sciences, Bhubaneswar, Bhubaneswar, IND

**Keywords:** anesthetic management, pulmonary alveolar proteinosis, dlt, one lung ventilation, whole lung lavage

## Abstract

Pulmonary alveolar proteinosis is an uncommon lung disease characterized by the accumulation of surfactant in the lungs. Treatment is done by whole lung lavage. One-lung ventilation in diseased lungs is a challenge to anesthesiologists due to the rapid desaturation and hemodynamic fluctuations encountered during the procedure. A 24-year-old female, a known patient of pulmonary alveolar proteinosis, who had undergone previous lung lavage presented with a dry cough and shortness of breath. Our management of the case included complete lung isolation with a double-lumen tube (DLT), one-lung ventilation, and an appropriate hemodynamic management strategy during the procedure.

## Introduction

Pulmonary alveolar proteinosis is a rare lung disease characterized by the accumulation of a lipoproteinaceous surfactant [1]. Most commonly, it may be autoimmune in origin or may result from mutation of surfactant genes, toxic inhalation, and hematological disorders. Whole lung lavage (WLL) is the mainstay of treatment in them [1]. Whole lung lavage is challenging to both the anesthesiologist and pulmonologist due to procedural complications like hemodynamic and oxygenation fluctuations. Here, we describe the safe anesthetic management of a young female with pulmonary alveolar proteinosis posted for whole lung lavage.

## Case presentation

**Figure 1:**
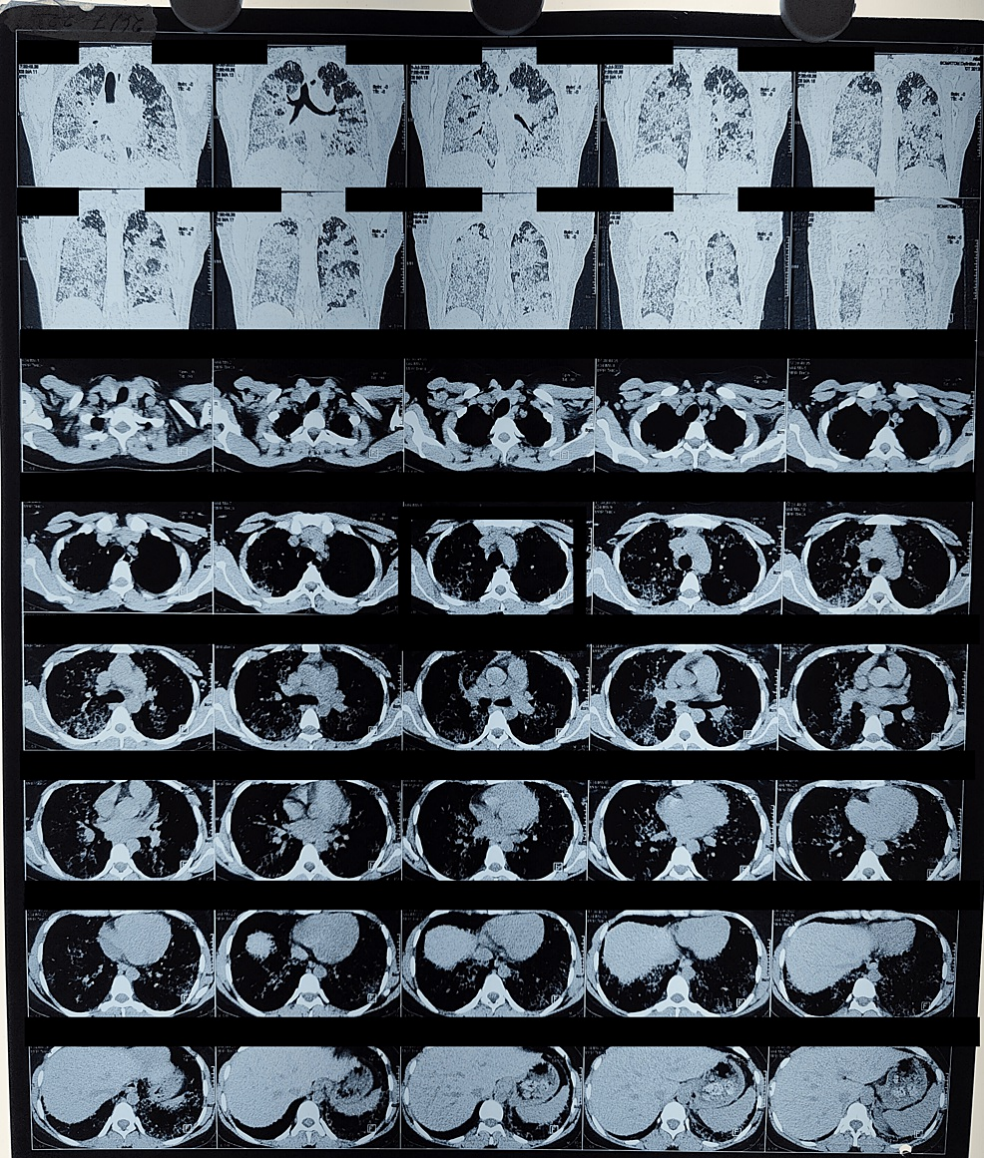
HRCT thorax HRCT: high-resolution computerized tomography

A 24-year-old female presented to pulmonology OPD with increasing shortness of breath and dry cough for the past month, which was associated with bilateral diffuse chest pain. She was a known patient of pulmonary alveolar proteinosis for the last four years and had undergone whole lung lavage four times previously. High-resolution computerized tomography (HRCT) showed ground glass opacities bilaterally with a crazy paving appearance (Figure 1).

Her previous lavage of the left lung was incomplete due to desaturation and hemodynamic changes. She was scheduled for whole lung lavage. On the day prior to surgery, informed and written consent was taken explaining the procedural complications, and she was advised alprazolam 0.25 mg at night and pantoprazole 40 mg on the morning of surgery.

On arrival at the operating room, ECG, pulse oximeter, and non-invasive blood pressure (NIBP) were attached, and her saturation was 92% on room air. 18G intravenous cannulation (left upper limb) was done and 20g radial artery cannulation was performed for continuous arterial pressure monitoring and serial arterial blood gas analysis. Anesthesia induction was done with 100 mcg of fentanyl and 70 mg of propofol, and muscle relaxation was facilitated with rocuronium 50 mg. After two minutes of ventilation, a 37Fr left-sided double-lumen endobronchial tube (DLT) was inserted and its position was confirmed by fiberoptic bronchoscope. Maintenance of anesthesia was done with 100% oxygenation and sevoflurane with volume-controlled ventilation. A 7Fr triple lumen central venous catheterization was done on the right internal jugular vein (IJV) under ultrasound guidance. As the previous lavage was unsuccessful on the left side, it was decided to first lavage the left lung followed by the right.

For lavage on the left side, the bronchial lumen was clamped and 100% oxygen was administered with low tidal volume and high respiratory rate. Lavage was started by the pulmonology team. Before lavage was started, a bubble test was done to rule out any leaks. Each cycle was done by instilling 500 ml aliquots of warm normal saline followed by drainage under gravity. In order to achieve maximal filling and drainage of all lung segments, manual chest vibration and percussion were done by the pulmonologist. Various positional maneuvers were also done to facilitate the run-in and run-out of fluid. There were episodes of desaturation (lowest saturation 80%) during drainage, which lasted for one minute, and improvement in saturation while instilling. Due to hemodynamic instability during the procedure, the patient was started on noradrenaline 0.05 mcg/kg/min to maintain blood pressure and dobutamine 3 mcg/kg/min to alleviate right heart failure. There was an increase in airway pressure and a decrease in tidal volume delivered associated with decreasing saturation level during lavage, which prompted us to check for fluid leakage. Fiberoptic bronchoscopic inspection and suction of the ventilated lung were done, which improved the tidal volume during subsequent cycles. After six cycles of lavage, there was clear fluid effluent.

**Figure 2:**
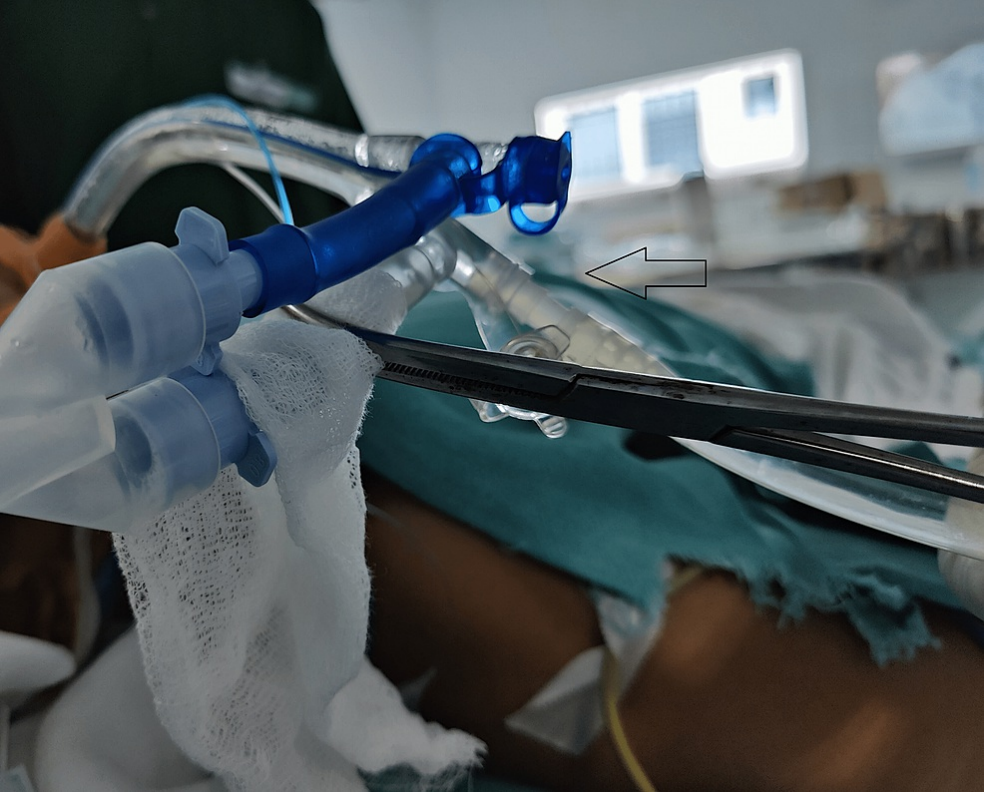
Lung isolation for right lung lavage

For lavage of the right lung, the bronchial lumen was unclamped and the tracheal lumen was clamped to initiate right lung lavage (Figure 2).

**Figure 3:**
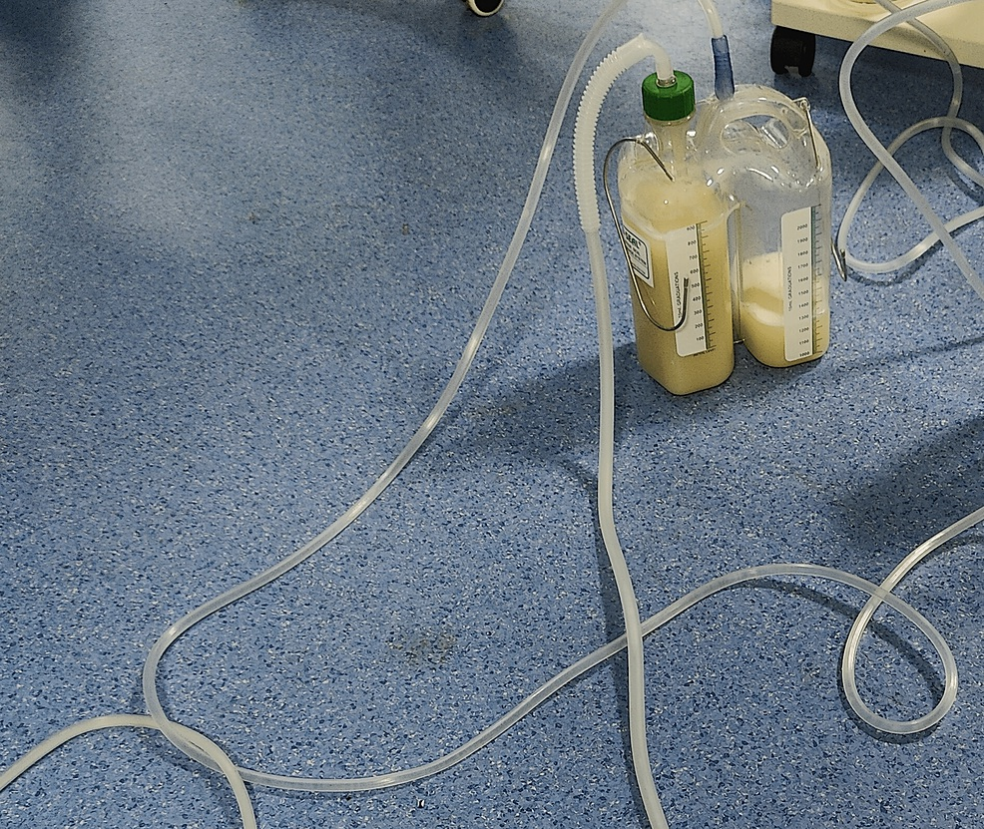
Lavage fluid

Seventeen cycles of lavage (Figure 3), each with 500 ml aliquots of warm normal saline, were done on the right lung, after which clear effluent was noted. This was better tolerated with well-maintained airway pressures and less desaturation (lowest saturation 88%), which lasted for less than 30 seconds probably due to improvement in the left lung.

At the end of the procedure, a double-lumen tube was exchanged with a 7.5 mm ID endotracheal tube, and the patient was shifted to the pulmonology ICU. She was given mechanical ventilatory support for 24 hours after which she was given a spontaneous breathing trial and was extubated. A post-procedure chest radiograph showed significant improvement.

## Discussion

Pulmonary alveolar proteinosis is a very rare disease with a prevalence approximating 3.7 cases per million per year [1]. Anesthetic management of a case of pulmonary alveolar proteinosis posted for whole lung lavage is very challenging. Complications commonly associated with the procedure include hypoxemia resulting in rapid desaturation and hemodynamic instability.

The main reason for hypoxemia is one-lung ventilation in a patient whose pulmonary function is already compromised. And there is a risk of spillage of lavage fluid to the ventilated lung which leads to an increase in peak airway pressures and decreased tidal volume. This can be detected by the appearance of bubbles in the water leak test done on the lavage side. If detected, bronchoscopic confirmation and rapid suctioning of the ventilated lung should be done to avoid a catastrophe [2]. Displacement of the DLT can also lead to loss of lung isolation and adequate lung isolation is the key to preventing spillage. And, episodes of desaturation are more common during the draining phase. So, positioning the ventilated lung in the dependent position during the draining phase may help in improving oxygenation [3]. Caution should be exercised, as this may lead to flooding of the dependent lung with fluid.

Various other strategies done for the management of hypoxemia during WLL include manual ventilation of a partially fluid-filled lung, intermittent double lung ventilation, and the use of inhaled nitric oxide [3,4]. Reports of hyperbaric oxygen and veno-venous extracorporeal membrane oxygenation (ECMO) in the management of WLL are present.

Hemodynamic instability may also occur due to a large amount of lavage fluid resulting in a mediastinal shift affecting the filling and ejection of the heart [2]. WLL is initially associated with an additional increase in pulmonary vascular resistance, greater than that resulting from single lung ventilation, which will cause the right ventricle to dilate [5]. So prior central venous catheter placement should be done to start inotropes and vasopressors if needed.

## Conclusions

To conclude, anesthetic management of whole lung lavage is very challenging. Cautious monitoring with complete isolation of the lung and the use of positional maneuvers to minimize desaturation and hemodynamic instability due to right ventricular dysfunction during the procedure and careful planning and management is vital in these cases.
