# Correction: Anesthetic Challenges During Whole Lung Lavage: A Case Report

**DOI:** 10.7759/cureus.c102

**Published:** 2023-02-17

**Authors:** Prasanta K Das, Krishna Prasanth Yadavilli

**Affiliations:** 1 Anesthesiology and Critical Care, All India Institute of Medical Sciences, Bhubaneswar, Bhubaneswar, IND; 2 Anaesthesiology, All India Institute of Medical Sciences, Bhubaneswar, Bhubaneswar, IND

This article has been corrected to replace Figure 1 with a version that properly obscures identifying patient information. This information was not detected prior to publication and the Cureus editorial department deeply regrets this oversight and error.

