# Correction: Anesthetic Challenges During Whole Lung Lavage: A Case Report

**DOI:** 10.7759/cureus.c103

**Published:** 2023-02-27

**Authors:** Prasanta K Das, Krishna Prasanth Yadavilli, Aswathy Girija, Subho Sarkar, Afshan Shaik, Manoj K Panigrahi

**Affiliations:** 1 Anesthesiology and Critical Care, All India Institute of Medical Sciences, Bhubaneswar, Bhubaneswar, IND; 2 Anaesthesiology, All India Institute of Medical Sciences, Bhubaneswar, Bhubaneswar, IND; 3 Pulmonary Medicine & Critical Care, All India Institute of Medical Sciences, Bhubaneswar, Bhubaneswar, IND

This article has been corrected to add the following authors who, due to miscommunication among the author group, were erroneously omitted from the original author list. The authors greatly regret that this error was not identified and resolved prior to submission and publication.

- Aswathy Girija
- Subho Sarkar
- Afshan Shaik
- Manoj K. Panigrahi

